# Uterine Angioleiomyoma with Atypia, Raised CA-125 Levels, and Pseudo-Meigs Syndrome: An Alarming Presentation

**DOI:** 10.1155/2012/519473

**Published:** 2012-05-30

**Authors:** Sunitha Thomas, Laxmi Radhakrishnan, Latha Abraham, Anna Matthai

**Affiliations:** Department of Pathology, Malakara Orthodox Syrian Church Medical College Hospital, Kolenchery, Ernakulam 682311, India

## Abstract

Angioleiomyomas are benign mesenchymal tumours commonly occurring in the subcutis of extremities. They are typically composed of interlacing fascicles of smooth muscle cells with intersecting vascular channels. Angioleiomyomas of the uterus are rare with only very few case reports available in literature. Herein, we report a case of this rare entity in a 47-year-old woman owing to its highly unusual features of cellular atypia, raised CA-125 levels, and pseudo Meigs syndrome.

## 1. Introduction

Angioleiomyoma is a rare and distinct variant of leiomyoma [1]. Uterine angioleiomyomas morphologically resemble those in the subcutis, the latter commonly occurring in the extremities, head, and trunk. Pseudo Meigs syndrome is a rare syndrome typically described with pelvic tumors (excluding ovarian fibromas) presenting with ascites and left pleural effusion. Pseudo Meigs syndrome and raised CA-125 levels have been rarely described in association with typical uterine leiomyomas. To the best of our knowledge, this is the first documented case of a uterine angioleiomyoma with atypia presenting with raised CA-125 levels and pseudo Meigs syndrome.

## 2. Case Report

A 47-year-old female presented to the gynaecology outpatient department with a one-year history of abdominal distension. She also had a history of menorrhagia, dyspepsia, and weight loss. On clinical examination, she was noted to have massive ascites with a palpable lower central abdominal mass. On vaginal examination, the uterus could not be felt separately from the mass. Her CA-125 level was 477.1 IU/mL (normal < 30 IU/mL). The possibility of an ovarian tumor or uterine sarcoma was raised clinically.

Ultrasound scan revealed a large mass posterior to the uterus along with massive ascites. CT scan performed showed a large predominantly solid pelvic mass demonstrating loss of fat plane with the uterine myometrium. The ovaries were not localized. There was massive ascites along with minimal left-sided pleural effusion. The diagnostic possibilities considered were those of a solid ovarian neoplasm infiltrating the uterus and a pedunculated uterine fibroid with sarcomatous change. The cytological examination of her ascitic fluid revealed no malignant cells.

 The patient underwent a total abdominal hysterectomy with bilateral salpingo-oophorectomy. About ten liters of serous ascitic fluid was drained. Preoperatively, a large irregular tumor with hemorrhage and degeneration was seen arising from the uterine isthmus.

**Figure 1:**
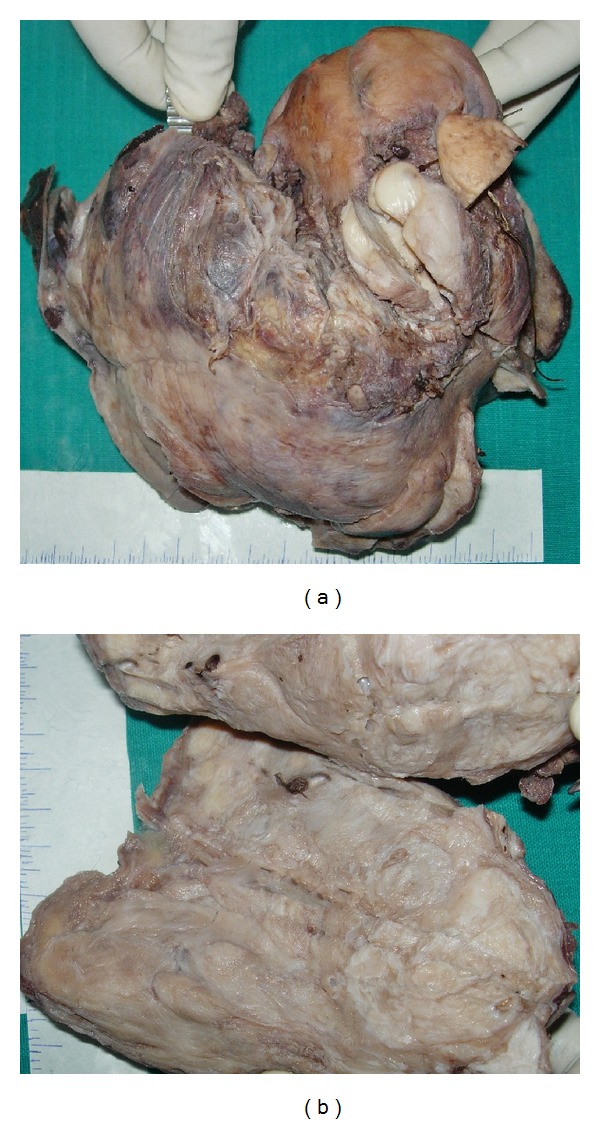
(a) Gross photograph showing a large hemorrhagic mass arising from the uterus. (b) Cut section of the tumor showing whitish whorled areas with interspersed blood vessels.

Grossly, the specimen with the attached tumor weighed 1.5 kgms. The uterus and cervix together measured 9 × 6 × 4 cm. A large hemorrhagic mass measuring 18 × 15 × 8 cm was seen to arise from the lower posterior part of the uterus (Figure 1(a)). On sectioning, the mass was noted to be in continuity with the myometrium. The cut surface of the mass was whitish and whorled (Figure 1(b)) with hemorrhagic areas in the periphery. Some dilated blood vessels with clots in their lumina were also present. The endometrium and the rest of the myometrium appeared unremarkable. Both ovaries and Fallopian tubes were normal. Postoperatively, the ascites resolved dramatically and her CA-125 levels normalized.

**Figure 2:**
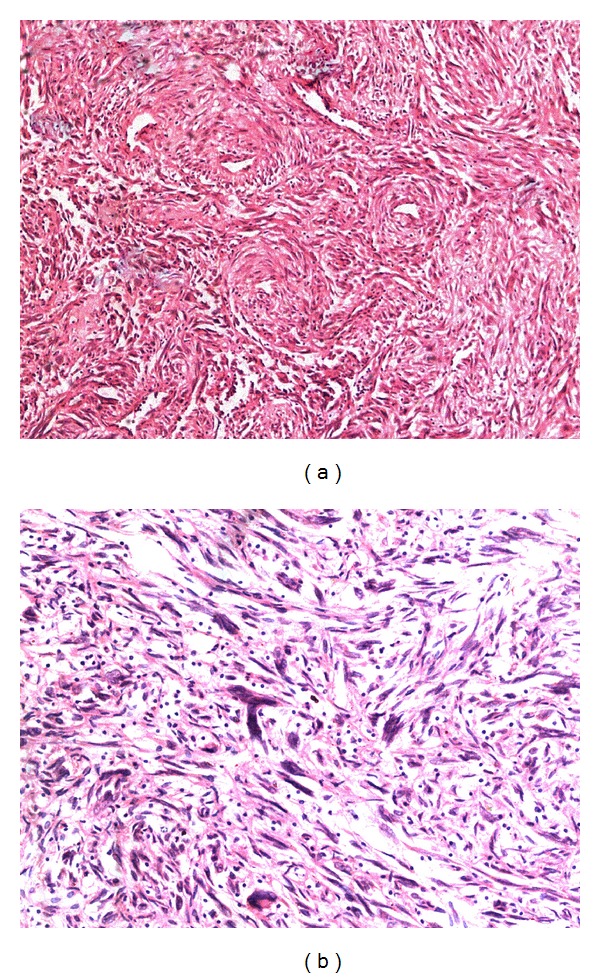
(a) Photomicrograph showing fascicles of spindle cells with interspersed abundant thick walled vessels. (b) Photomicrograph showing spindle cells with marked cellular atypia.

Histological examination of the tumor showed a moderately cellular neoplasm composed of interlacing fascicles of spindle cells with interspersed abundant thick muscular walled vessels (Figure 2(a)). Bland spindle cells were seen swirling around some of the vessels. In areas, the spindle cells showed marked atypia with bizarre hyperchromatic nuclei (Figure 2(b)) and some multinucleate giant cells. However, extensive sampling revealed no excess or atypical mitotic activity, coagulative tumor cell necrosis, or hypercellularity. There was subcapsular and intratumoral hemorrhage along with foci of hyalinisation and edema. The endometrium, bilateral tubes, and ovaries revealed no significant pathology.

**Figure 3:**
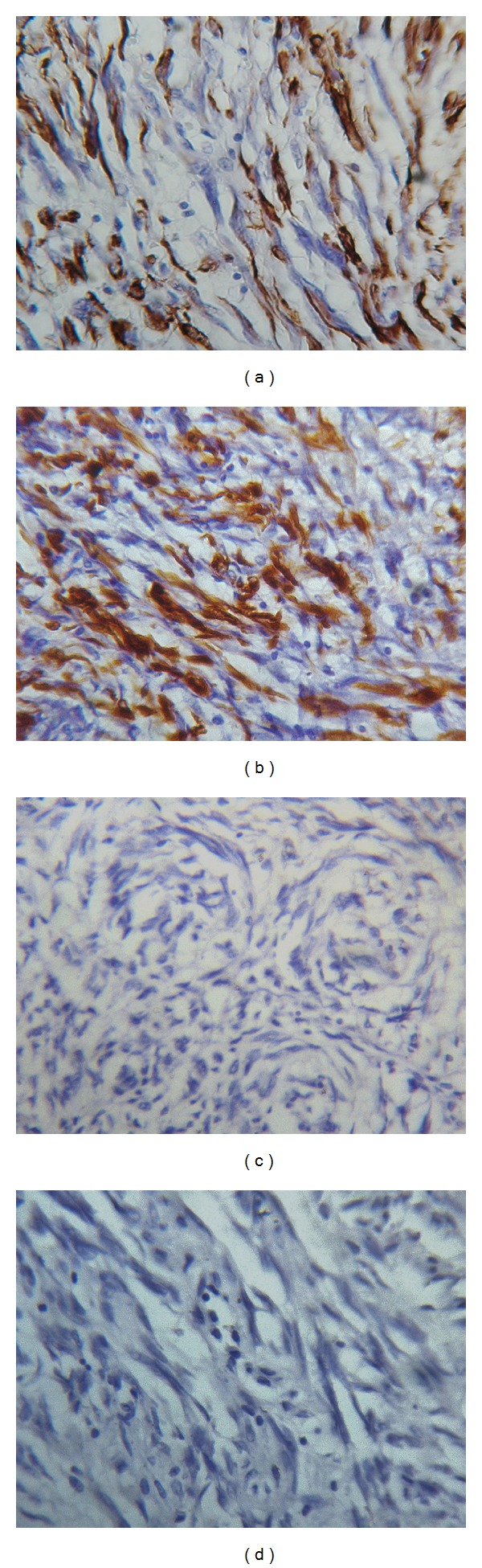
(a) Smooth muscle actin. (b) desmin. (c) HMB 45. (d) Ki 67.

On immunohistochemistry, the tumor cells showed positivity for smooth muscle actin (SMA) (Figure 3(a)) and desmin (Figure 3(b)). HMB-45 negativity (Figure 3(c)) ruled out the possibility of a perivascular epithelioid cell tumor (PEComa). The Ki-67 proliferation index (Figure 3(d)) was less than 1%.

The patient has had no complications or further symptoms on followup for 6 months post-op and to date.

## 3. Discussion

Angioleiomyomas are benign, relatively common neoplasms described in the lower extremities, head, and trunk. However, only very few cases of angioleiomyomas have been described in the uterus [2] and they represent a subtype of the uterine leiomyoma. They occur usually in the fourth to sixth decades [3] and can present as an abdominal mass or with symptoms of abdominal pain and menorrhagia [4]. These tumors can undergo spontaneous rupture and cause catastrophic intraabdominal bleeding [3]. Similar to angioleiomyomas elsewhere, uterine angioleiomyomas are composed of smooth muscle bundles with prominent thick walled blood vessels. Three histological subtypes have been recognised: solid, venous, and cavernous [5].

Kawagishi et al. [6] and J. A. Martínez et al. [7] have reported cases of pleomorphic angioleiomyomas in the subcutis of lower extremities and scrotum. In both these reports, there was marked nuclear pleomorphism; however, no mitotic figures were identified. An extensive literature review performed did not reveal any reports on uterine angioleiomyomas with pleomorphism. Our case exhibited foci of marked nuclear atypia. Thorough sampling of the tumor did not reveal coagulative necrosis or mitotic activity, and the Ki-67 proliferation index was less than 1%.

CA-125 is a valuable serum marker that aids in the diagnosis of ovarian cancer. However, CA-125 levels are not entirely specific for ovarian carcinomas and can be seen elevated in several nonneoplastic and other neoplastic conditions including benign ovarian fibromas [8] and uterine leiomyomas [9]. The increased level of CA 125 most probably resulted from the peritoneal mechanical irritation from the large pelvic mass or from a large volume of ascites.

Pseudo Meigs syndrome encompasses the clinical triad of hydrothorax, ascites, and pelvic tumours/ovarian tumours (excluding ovarian fibromas). In contrast to Meigs syndrome, which has been typically described in association with benign ovarian fibromas, pseudo Meigs syndrome can be seen with benign and malignant tumours including uterine leiomyomas [10], leiomyomas of the broad ligament and malignant ovarian tumours including primary tumours like adenocarcinomas and endometrioid carcinomas and secondary metastatic tumours to the ovary. Ours is the first described case of a uterine angioleiomyoma with atypia associated with raised CA-125 levels and pseudo Meigs syndrome.

The study on vascular system of intramural leiomyomas by Walocha et al. [11] revealed that usual leiomyomas contained vascular network with density similar to or lower than that of normal myometrium. These are predominantly capillaries along with a few arterioles and small arteries. In contrast angioleiomyomas have abundant thick walled vessels with intersecting smooth muscle bundles.

One of the differential diagnoses considered in this case was a PEComa. In our case, the tumor cells showed perivascular accentuation around thick walled vessels but lacked the clear or eosinophilic granular cytoplasm described in the latter. The cells were negative for HMB-45 and positive for SMA and desmin, thereby ruling out the possibility of a PEComa. In cases of angioleiomyomas that exhibit atypia, the possibility of a leiomyosarcoma should be excluded. This case illustrates the importance of thorough sampling of such tumors to identify foci of coagulative tumor cell necrosis, increased mitotic activity, and atypical mitoses, which are diagnostic features of leiomyosarcoma. The other differential diagnosis includes symplastic leiomyoma, which also exhibits cellular atypia but lacks abundant thick walled blood vessels with perivascular spindle cell swirling.

In conclusion, this is a unique case of a uterine angioleiomyoma with atypia in a middle-aged woman who presented with clinically worrisome massive ascites and markedly elevated CA-125 levels. Atypia of tumor cells in such a clinical context can be alarming, but diligent sampling and histopathological examination of these tumors can provide crucial diagnostic pointers to the benign nature of these lesions.
